# Anatomical and functional changes in the retina in patients with Alzheimer’s disease and mild cognitive impairment

**DOI:** 10.1111/aos.14419

**Published:** 2020-03-25

**Authors:** Stephan Szegedi, Peter Dal‐Bianco, Elisabeth Stögmann, Tatjana Traub‐Weidinger, Michael Rainer, Andreas Masching, Doreen Schmidl, René M. Werkmeister, Jacqueline Chua, Leopold Schmetterer, Gerhard Garhöfer

**Affiliations:** ^1^ Department of Clinical Pharmacology Medical University of Vienna Vienna Austria; ^2^ Department of Neurology Medical University of Vienna Vienna Austria; ^3^ Division of Nuclear Medicine Department of Biomedical Imaging and Image‐guided Therapy Medical University of Vienna Vienna Austria; ^4^ Department of Psychiatry Social and Medical Centre East – Danube Hospital Vienna Austria; ^5^ Karl Landsteiner Institute for Memory and Alzheimer Research Vienna Austria; ^6^ Center for Medical Physics and Biomedical Engineering Medical University of Vienna Vienna Austria; ^7^ Singapore Eye Research Institute Singapore National Eye Centre Singapore City Singapore; ^8^ Ophthalmology and Visual Sciences Academic Clinical Program Duke‐NUS Medical School Singapore City Singapore; ^9^ Lee Kong Chian School of Medicine Nanyang Technological University Singapore City Singapore; ^10^ Institute of Molecular and Clinical Ophthalmology Basel Switzerland

**Keywords:** Alzheimer’s disease, mild cognitive impairment, oxygen, retinal blood flow

## Abstract

**Purpose:**

There is evidence that mild cognitive impairment (MCI) or Alzheimer's disease (AD) is accompanied by alterations in the retina. The current study was performed to investigate structural and functional changes in patients with systemic neurodegenerative disease.

**Methods:**

A total of 47 patients with either MCI or AD and 43 healthy age‐ and sex‐matched control subjects were included. Inclusion criteria for MCI were abnormal memory function and a mini‐mental state examination (MMSE) score >26 points, for patients with AD a diagnosis of probable AD of mild to moderate degree and an MMSE score in the range of 20–26. Retinal blood flow was measured using a Doppler optical coherence tomography (OCT) system. Retinal vessel diameter, oxygen saturation and flicker‐induced vasodilatation were measured using a Vessel Analyzer. Retinal nerve fibre layer thickness (RNFLT) was assessed using an OCT system.

**Results:**

Global RNFLT was lower in patients compared to healthy controls (93.7 ± 12.8 µm versus 99.1 ± 9.0 µm, p = 0.02). The same was found in regards to retinal arterial blood flow, which was 9.3 ± 2.4 and 12.3 ± 3.2 μl/min in the patient and control groups, respectively (p < 0.001). Mean retinal arterial diameter was reduced in patients (76.0 ± 8.9 µm versus 80.6 ± 8.0 µm, p = 0.03). Arteriovenous difference in oxygen saturation was lower in patients (20.4 ± 5.1% versus 23.5 ± 4.0%, p < 0.01). No difference in the flicker response was observed.

**Conclusion:**

In patients with MCI and AD, arteriovenous difference in oxygen saturation, retinal blood flow and arterial vessel diameter was reduced. No difference was found in flicker response between groups. This indicates alterations in retinal oxygen metabolism in patients with neurodegenerative disease.

## Introduction

Alzheimer’s disease (AD) is a major cause of dementia and a considerable healthcare problem worldwide (Ferri et al. 2005). Despite many efforts, the exact pathogenesis of AD is not yet fully clarified and the primary cause of the disease is only poorly understood. Briefly, according to the most widely accepted model for AD, accumulation of amyloid‐beta peptide (Aβ) in brain tissues is one of the key events in the pathogenesis of AD. Clinically, these neurodegenerative processes can be identified by a progressive decrease in memory, learning and executive function. As a transitional stage between normal ageing and dementia, ‘mild cognitive impairment’ (MCI) may precede the full clinical picture of AD. However, the transition from MCI to AD is fluid, and a definite diagnosis of AD can only be confirmed post‐mortem (Boccardi et al. 2017). In this context, there is evidence that patients with MCI have a high risk of developing dementia, in particular AD.

The absence of easily usable biomarkers for the diagnosis and the clinical monitoring of AD (Henriksen et al. 2014) has stimulated research in other areas of the body, among which the eye offers several considerable advantages for detection and follow‐up of neurodegenerative processes (Frost et al. 2010). In particular, due to its unique optical properties, the eye allows for non‐invasive imaging of neural tissue as well as its microcirculation with an unprecedented high resolution up to micrometre range. Indeed, data from previously published studies indicate that structural alterations (Liu et al. 2015) of the retina are present in patients with AD (Cunha et al. 2016; Mutlu et al. 2018; Asanad et al. 2019; Cipollini et al. 2019). However, whether these structural changes may be suitable as a biomarker for early‐stage disease is still a matter of controversy (van de Kreeke et al. 2019a).

Beside these structural changes, reduced cerebral blood flow has been discussed as a potential pathogenic factor for the development of the disease and may therefore be a potential biomarker for progression of AD (Ruitenberg et al. 2005). It is well accepted now that AD is associated with morphological as well as functional alterations of cerebral blood vessels and the perivascular neuronal tissue (Ries et al. 1993; Schuff et al. 2009; Snyder et al. 2014). Further, there is strong evidence that in patients with AD the coupling between neural activity and blood flow (neurovascular coupling) is perturbed in close association with cognitive decline (Lourenco et al. 2015).

There is increasing evidence that these vascular changes are also reflected in the retina. Data from optical coherence tomography (OCT) angiography studies show alterations in the retinal microcirculation of subjects with preclinical and manifest AD (Grewal et al. 2018; O'Bryhim et al. 2018; Yoon et al. 2019). Further evidence from a small‐scale study indicates possible alterations in ocular blood flow in patients with AD (Berisha et al. 2007). New imaging modalities such as Doppler OCT systems allow not only for *in vivo* imaging of anatomical structures, but also allow for quantification of functional measurements such as real time‐blood flow (Leitgeb et al. 2014). Further, imaging‐based measurement of retinal oxygen saturation makes it possible to obtain insight into metabolic changes on the level of the retina, which in turn may increase our understanding of the pathophysiology of neurodegenerative diseases (Stefansson et al. 2019).

The current study was designed to investigate possible changes in the ocular microcirculation and oxygen metabolism in patients with AD and MCI. For this purpose, a custom‐built dual‐beam Doppler OCT system for blood flow measurement in the retina was used, allowing for the assessment of volumetric blood flow in this group of patients. Further, neurovascular coupling was measured using the hyperaemic response of retinal vessels to flicker light stimulation as published previously (Garhofer et al. 2002; Garhofer et al. 2004; Riva et al. 2005; Kur et al. 2012; Newman 2013). Finally, retinal oxygenation was measured using reflectometry. Anatomy of the posterior eye pole was assessed using OCT. Patients with AD and MCI were included and compared with age‐matched healthy control subjects. We tested the hypothesis that ocular blood flow and metabolic autoregulation are altered in patients with AD and MCI.

## Research Design and Methods

### Subjects

The study was approved by the Ethics Committee of the Medical University of Vienna and followed the guidelines set forth in the Declaration of Helsinki. Patients with MCI and AD were recruited by specialized Memory Clinics in Vienna, Austria and then referred to the study centre. Diagnosis was made by fully trained neurologists/psychiatrists (PD, ES, MR and AM). Healthy subjects were recruited at the Department of Clinical Pharmacology. Subjects were only included after having provided written informed consent following a detailed explanation of the study procedures. Before the study, subjects passed a screening examination that included physical examination, mini‐mental state examination (MMSE), assessment of visual acuity, slit lamp biomicroscopy, funduscopy and measurement of intraocular pressure (IOP) using Goldmann applanation tonometry.

Inclusion criteria for patients with AD were confirmed diagnosis of AD of mild to moderate degree based on the National Institute of Neurological and Communicative Disorders and Stroke–Alzheimer’s Disease and Related Disorders Association (NINCDS/ADRDA) criteria and MMSE score between 20 and 26 points. Mild cognitive impairment (MCI) was defined as memory complaint, corroborated by an informant, abnormal memory function, documented by delayed recall of one paragraph from the Logical Memory II subtest of the Wechsler Memory Scale‐Revised, MMSE score >26 points and being not sufficiently impaired to meet the NINCDS/ADRDA criteria, as judged by an experienced AD research clinician. The Hachinski Ischemia Scale was used to distinguish AD and MCI from multi‐infarct dementia.

Ophthalmic inclusion criteria were visual acuity of 20/40 or better, IOP < 21 mmHg and normal findings from slit lamp biomicroscopy and funduscopy. Patients were only included if no abnormalities in the ophthalmic examination were found. Exclusion criteria were ametropia ≥6 dioptres, untreated arterial hypertension, history or family history of epilepsy, and any clinically relevant illness as judged by the investigators. Participants had to abstain from beverages containing alcohol or xanthine derivates in the 12 hr before the study day.

### Protocol

The study eye was dilated with a drop of tropicamide (Mydriatikum AGEPHA, Vienna, Austria). This was followed by a resting period of at least 20 min to ensure full development of mydriasis and stabilization of blood pressure. Thereafter, retinal blood flow was measured using the custom‐built dual‐beam bidirectional Doppler Fourier‐Domain OCT. Fundus photographs were taken using a Retinal Vessel Analyzer (RVA; Imedos Systems UG, Jena, Germany) in order to quantify the retinal oxygen saturation. Blood pressure and pulse rate were measured non‐invasively.

### Measurement of intraocular pressure, blood pressure and pulse rate

Intraocular pressure was measured using a Goldman applanation tonometer. Systolic, diastolic and mean arterial blood pressures (SBP, DBP and MAP) were measured on the upper arm by an automated oscillometric device (Infinity Delta, Dräger, Vienna, Austria). The same device was used to record pulse rate. Ocular perfusion pressure (OPP) in the sitting position was calculated as OPP = 2/3*MAP − IOP (Costa et al. 2014).

### Measurement of RNFL and central retinal thickness

Structural OCTs were recorded using a commercially available spectral‐domain OCT system (SD‐OCT, Heidelberg Spectralis OCT, SPECTRALIS software version 5.3.3.0, EYE EXPLORER Software 1.6.4.0; Heidelberg Engineering, Heidelberg, Germany). A circular scan with a 3.46 mm‐diameter centred on the optic disc was performed and RNFL thickness values were provided for four quadrants, six sectors and an overall average over the entire 360 degrees. These average RNFL thickness measurements derived from SPECTRALIS RNFL analysis report were used for the present study. In addition, central retinal thickness was assessed using the standard scanning protocol for the macula (macular volume scans, 20° × 20° with 97 horizontal sections).

### Measurement of retinal blood flow

For the measurement of retinal blood flow, we used a custom‐built dual‐beam Doppler Fourier‐Domain OCT system coupled to a fundus camera. This system has been described in detail previously (Doblhoff‐Dier et al. 2014). With this system, we illuminate the vessels with two orthogonally polarized probe beams that emanate from the same superluminescent diode under a defined angle Δ∝ to overcome the problem of angle‐ambiguity. Two spectrometers collect the spectral information of the two orthogonally polarized laser beams and two OCT images are formed under two different angles of incidence. The phase changes in these channels are different according to the different Doppler angles. The central wavelength of the laser is at $\lambda_{0}$ = 838.8 nm with a spectral bandwidth of 54.0 nm. This results in an axial resolution in tissue of approximately 6 µm. The lateral resolution of the system is approximately 20 µm.

We have previously shown how to quantify absolute blood velocity (Werkmeister et al. 2008; Werkmeister et al. 2012a; Werkmeister et al. 2012b). The two probing beams are characterized by the wave vectors $\vec{\kappa_{1}}$ and $\vec{\kappa_{2}}$ and illuminate the red blood cells that move along velocity vector $\vec{\nu }$ . Scattering of the individual probe beam lights at the moving red blood cells induces a phase shift between two subsequent A‐scans recordings

(1) $$\Phi_{i}^{\prime }=2\vec{K_{i}}\vec{\nu_{i}}\tau \;\left(i=1.2\right).$$

In this equation, *τ* is the time span between two subsequent recordings, which equals the A‐scan rate of the system. We have previously shown that the absolute velocity (*V*_abs_) of the blood in the vessel can be calculated as

(2) $$V_{\text{abs}}=\Delta \Phi \frac{\lambda }{4\pi \cdot n\cdot \Delta a\cdot \cos\beta }$$

As long as Δα is small. In this equation $\Delta \Phi =\Phi_{1}-\Phi_{2}$ , *λ* is the central wavelength of the light source, *β* is the angle between the plane spanned by $\vec{\kappa_{1}}$ and $\vec{\kappa_{2}}$ and the velocity vector, and *n* is the group refractive index of blood.

In the present study, we have used a slightly different novel approach to calculate the absolute blood velocity. The phase shifts in the two channels can be written as follows (Werkmeister et al. 2012a):

(3) $$\Phi_{1}=V_{\text{abs}}\cdot \left(\frac{4\pi n\tau \cos\beta }{\lambda }\right)\cdot \left(\frac{\pi }{2}-\varepsilon \right)$$

(4) $$\Phi_{2}=V_{\text{abs}}\cdot \left(\frac{4\pi n\tau \cos\beta }{\lambda }\right)\cdot \left(\frac{\pi }{2}-\varepsilon -\Delta \alpha \right)$$

In this equation, the angle *ε* is given by $\alpha_{1}-\gamma$ , where *γ* is the angle between the velocity vector and the plane perpendicular to the optical axis of the illuminating beam. If *V*_abs_ is calculated according to eqn 2, two values of *ε* can be calculated from eqns 3 and 4, respectively, called *ε*_1_ and *ε*_2_. When the angle *ε*_1_ and *ε*_2_ is calculated, eqn (1) allows for calculation of *V*_abs,1_ and *V*_abs,2_. We have previously used these calculations for checking the quality of our measurements. In the present study, we used a different approach and calculated *V* as $\frac{V_{\text{abs}.1}+V_{\text{abs}.2}}{2}$ . In addition, data were only considered as valid when the difference between *V*_1_ and *V*_2_ was less than 25%. All other data were not considered as reliable. We have shown in healthy subjects that *V* shows better reproducibility and repeatability than *V*_abs_ (intraclass correlation coefficient 0.89 versus 0.81, unpublished data). As such, *V* was used to calculate blood flow in retinal arteries and veins. The advantage of this novel was to calculate absolute blood velocity is that it is less sensitive to poor signal quality in one of the channels. In addition, it introduces on objective criterion for quality control.

In addition, the diameter of the vessels (d) needs to be calculated. For this, we followed an approach described in Fondi et al. (2016) that uses the phase image for measuring vessel calibre. Hence, blood flow in arteries and veins was calculated as $Q_{A}=V_{A}\cdot d_{A}^{2}\cdot \frac{\pi }{4}$ and $Q_{V}=V_{V}\cdot d_{V}^{2}\cdot \frac{\pi }{4}$ (Fondi et al. 2016).

In all study participants, measurements were obtained from one major temporal retinal artery and vein approximately one disc diameter from the ONH.

### Measurement of flicker‐induced vasodilatation

For the assessment of flicker‐induced vasodilatation, a commercially available Dynamic Vessel Analyzer (DVA, Imedos, Jena, Germany) was used. This device comprises a fundus camera, a video camera, a real‐time monitor and a personal computer with an analysing software for the determination of retinal arterial and venous diameters (Blum et al. 1999). Each second, a maximum of 25 readings of vessel diameter can be obtained. The DVA has a built‐in flicker stimulation system that generates a square wave stimulation pattern with a frequency of 12.5 Hz and a modulation depth of 100% (Garhofer et al. 2010). Vessel diameter measurements were performed for 60 seconds at baseline (BL) and for 60 seconds during flicker (FL) stimulation. Due to the limited attention span of the patients, measurements were only performed once with no repetition.

### Measurement of retinal oxygen saturation and vessel diameter

For assessment of retinal oxygen saturation, the oxygen module of the DVA was used. Its software allows for the measurement of retinal oxygen saturation from fundus photographs based on reflectance spectroscopy with a two‐wavelength method (Hammer et al. 2008). Measurements were performed from 50 degree images in all vessels in a peripapillary annulus with an inner radius of 1 and an outer radius of 1.5 disc diameters. For analysis, the image with the highest quality was used. Oxygen saturation and diameter values for all retinal arteries and veins were averaged.

### Data analysis

All outcome parameters were checked for normality using the Shapiro–Wilk test. Un‐paired t‐tests were used to compare groups. Flicker‐induced vasodilatation was calculated as per cent change over baseline. In addition, correlation analyses were performed for global RNFL thickness as a structural parameter with vascular functional parameters (retinal blood flow, retinal oxygen saturation and flicker‐induced vasodilatation). Data are presented as means ± SD. A p‐value < 0.05 was considered the level of significance. Statistical analysis was carried out using CSS Statistica for Windows^®^ (Statsoft Inc., version 6.0, Tulsa, CA, USA).

## Results

A total of 90 subjects were included in the present study, of which 47 were patients with either a diagnosis of AD (*n* = 23) or MCI (*n* = 24) and 43 were healthy control subjects. All subjects were of Caucasian ancestry. A demographic overview is given in Table 1. No differences in age, sex, MAP, IOP and OPP were observed. As expected, MMSE was significantly lower in the patient group compared to the healthy control subjects, which all passed with the maximum score of 30 points.

**Table 1:** Baseline characteristics of the two study groups for mean arterial pressure (MAP), intraocular pressure (IOP), ocular perfusion pressure (OPP) and mini‐mental state examination (MMSE).

	AD/MCI	Healthy controls	p‐Value
Age (years)	73.4 ± 8.9	70.7 ± 6.9	0.11
Sex (f/m)	19/28	23/20	0.21
MAP (mmHg)	104.9 ± 10.8	102.2 ± 9.8	0.24
IOP (mmHg)	15.1 ± 2.9	14.8 ± 2.5	0.62
OPP (mmHg)	54.9 ± 7.4	53.3 ± 6.4	0.31
MMSE (points)	25 ± 3	30 ± 0	<0.001

AD = Alzheimer's disease, MCI = mild cognitive impairment.

All values are given as means ± SD.

In both groups, several subjects were taking antihypertensive medication as shown in Table 2. The distribution of the different classes of antihypertensive drugs was similar between groups.

**Table 2:** Number and percentage of subjects taking any concomitant antihypertensive medication, also listed according to substance classes.

	AD/MCI	Healthy controls	p‐Value
Any antihypertensive concomitant medication	23/47 (48.9%)	19/43 (44.2%)	0.66
Alpha receptor antagonists	0/47 (0.0%)	1/43 (2.3%)	0.30
ACE inhibitors	8/47 (17.0%)	7/43 (16.3%)	0.93
Angiotensin receptor antagonists	10/47 (21.3%)	5/43 (11.6%)	0.22
Beta blockers	6/47 (12.8%)	9/43 (20.9%)	0.30
Calcium channel blockers	6/47 (12.8%)	4/43 (9.3%)	0.61
Diuretics	5/47 (10.6%)	5/43 (11.6%)	0.88
Renin inhibitors	0/47 (0.0%)	1/43 (2.3%)	0.30
Vasodilators	1/47 (2.1%)	1/43 (2.3%)	0.95

AD = Alzheimer's disease, MCI = mild cognitive impairment.

Some subjects took more than one substance.

**Fig. 1:**
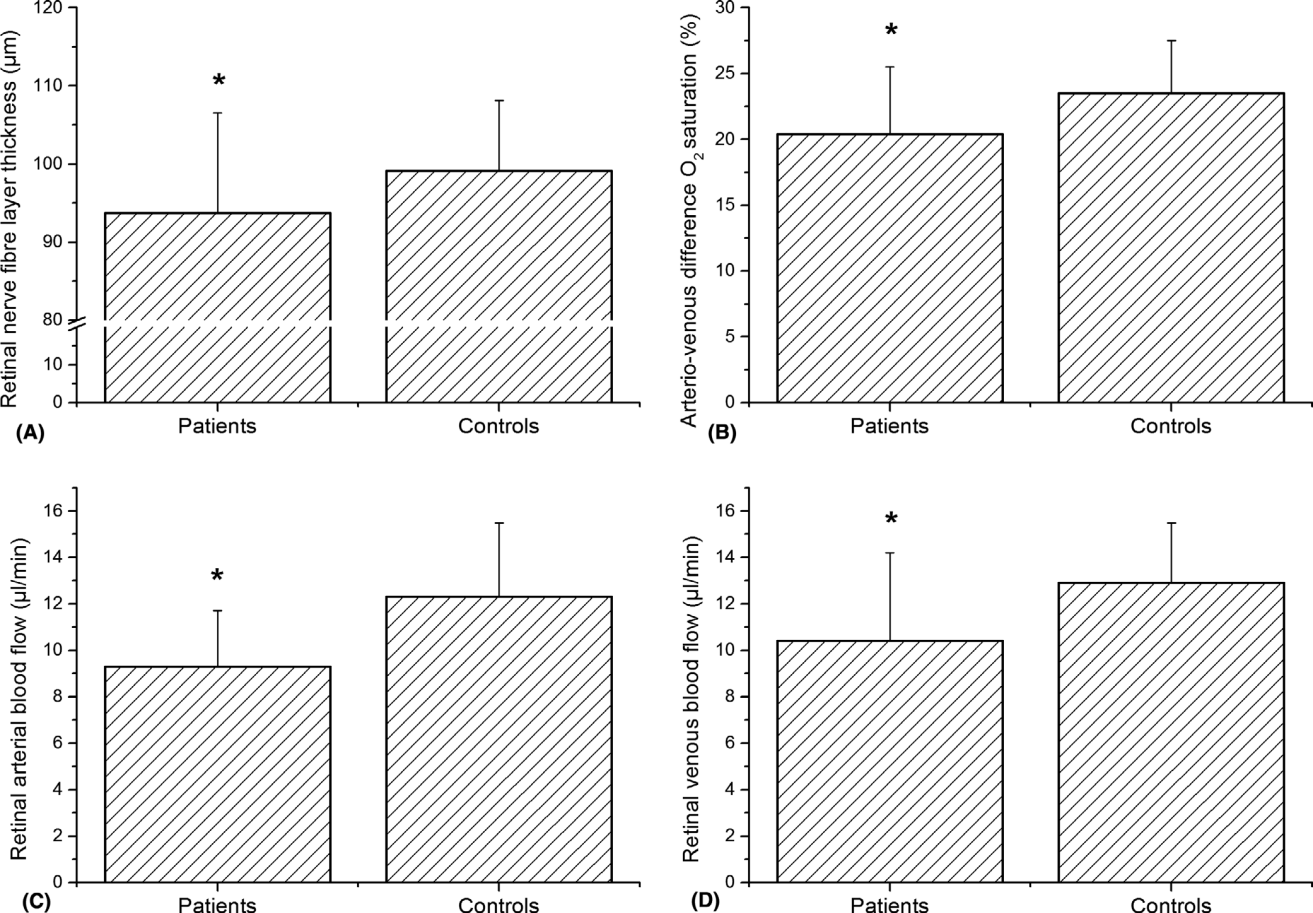
Differences in global retinal nerve fibre layer thickness (A), retinal arterial blood flow (B), retinal venous blood flow (C) and arteriovenous difference in oxygen saturation (D) between patients with Alzheimer’s/mild cognitive impairment (MCI) and healthy controls. All values are presented as means ± SD. *Significant between groups.

Global RNFL thickness was significantly lower in patients compared to healthy controls (93.7 ± 12.8 µm versus 99.1 ± 9.0 µm, p = 0.02, Fig. 1A). This effect was most pronounced in the temporal superior and the temporal sector. No significant difference was found between the other sectors or central retinal thickness as obtained from the macular scan, although all values tended to be lower in the patient group. Table 3 shows values for RNFL for all sectors and for central retinal thickness.

**Table 3:** Retinal nerve fibre layer thickness (RNFLT) in all sectors for both groups and central retinal thickness obtained from the macular scan (CRT).

	AD/MCI	Healthy controls	p‐Value
RNFLT			
Global	93.7 ± 12.8	99.1 ± 9.0	0.02
Temporal superior	122.3 ± 23.2	133.1 ± 17.8	0.02
Nasal superior	108.3 ± 25.2	112.5 ± 21.6	0.40
Nasal	76.2 ± 12.4	79.3 ± 17.2	0.33
Nasal inferior	107.4 ± 27.2	113.7 ± 26.7	0.27
Temporal inferior	140.1 ± 26.6	149.1 ± 21.1	0.08
Temporal	68.7 ± 13.3	75.8 ± 16.1	0.03
CRT	266.9 ± 22.5	277.5 ± 22.5	0.08

AD = Alzheimer's disease, MCI = mild cognitive impairment.

All values are given in µm and shown as means ± SD.

According to the criteria in the methods section, retinal blood flow could be quantified in 30 patients with AD/MCI and in 29 healthy control subjects. Both *Q*_A_ and *Q*_V_ were lower in the group of patients. *Q*_A_ was 9.3 ± 2.4 and 12.3 ± 3.2 μl/min in the patient and control group, respectively (p < 0.001, Fig. 1B). In retinal veins, the difference was less pronounced with values of *Q*_V_ _=_ 10.4 ± 3.8 μl/min in patients with AD/MCI and *Q*_V_ _=_ 12.9 ± 2.6 μl/min in control subjects (Fig. 1C).

Mean retinal arterial diameter as obtained from the DVA measurements was significantly reduced in patients compared to controls (76.0 ± 8.9 µm versus 80.6 ± 8.0 µm, p = 0.03), while no difference in venous diameter was found between groups (104.6 ± 13.2 µm versus 107.8 ± 9.9 µm, p = 0.27).

While retinal arterial oxygen saturation was similar between groups (94.5 ± 4.7% in patients and 94.5 ± 4.0% in healthy controls, p = 0.99), venous oxygen saturation was significantly higher in patients compared to controls (74.1 ± 7.2% versus 71.0 ± 5.2%, p = 0.04). This is reflected in the arteriovenous difference in oxygen saturation, which was found to be significantly lower in patients with neurodegenerative disease compared to healthy controls (20.4 ± 5.1% versus 23.5 ± 4.0%, p < 0.01, Fig. 1D).

Stimulation with flicker light induced an increase in retinal arterial diameter of +2.1 ± 2.6% in patients and of +2.4 ± 2.6% in healthy controls (p = 0.71 between groups). In retinal veins, an increase of +3.5 ± 2.1% and of +3.7 ± 2.5% was seen in patients and controls, respectively (p = 0.63 between groups).

Correlation analysis between global RNFL thickness and the vascular functional parameters such as flicker response, retinal blood flow and A‐V difference, revealed no statistically significant correlations (data shown in Table S1).

## Discussion

Our study demonstrates a decrease in retinal blood flow in patients with MCI and AD using a custom‐built dual‐beam Doppler OCT system to assess volumetric blood flow. This decrease in retinal blood flow is accompanied by a significant thinning in RNFL thickness and by a reduction of retinal‐venous oxygen difference. Thus, our results show that beside structural changes, patients with AD and MCI show reduced retinal metabolism compared healthy age‐matched subjects. However, no correlation between structural measures and vascular functional parameters such as blood flow or oxygen saturation was observed. Further, no difference was found in flicker‐induced vasodilatation between healthy subjects and the patient group.

There is a longstanding discussion whether, and if so, to which degree cerebral pathologies associated with AD are reflected in the retina of MCI and AD patients. First histological studies more than 30 years ago reported anatomical alterations such as a reduction in the number of ganglion cells and RNFL thickness in post‐mortem eyes of patients with AD compared to healthy subjects (Hinton et al. 1986). Using non‐invasive techniques such as OCT, several *in vivo* studies found anatomical changes including reduction of RNFL thickness in patients with preclinical and manifest AD (Cunha et al. 2016; Snyder et al. 2016; Asanad et al. 2019; Cipollini et al. 2019). This is also supported by the findings of the present study, where a global reduction in RNFL thickness was found in patients with AD and MCI. This decline was more pronounced in the temporal retinal sector, although a tendency towards a decrease in RNFL thickness was found in all four quadrants. Whether this can be interpreted as a higher susceptibility of the temporal sectors because of higher metabolic turnover is unclear and warrants further investigation. However, recently doubts have been raised whether retinal thickness alone is a suitable parameter for the early identification of preclinical AD patients (van de Kreeke et al. 2019a). Thus, it has been hypothesized that functional parameters may be more sensitive than structural parameters alone (Liao et al. 2018).

The results of the current study show a reduced arteriovenous oxygen difference accompanied by a lower retinal blood flow in patients with AD and MCI compared to age‐matched, healthy control subjects, indicating for reduced retinal metabolism in patients with cognitive impairment. Further, our data indicate that retinal arterial diameters are reduced in the disease groups. Although only few studies have investigated possible alterations in the retinal microcirculation of the eye of patients with AD or MCI, our findings are well in line with the data from a previous experiment. As such, a small‐scale study using laser Doppler flowmetry indicated reduced blood flow patients with AD (Berisha et al. 2007). However, this study only included nine AD patients, and the general applicability of the results is limited. Further, no data are available from the latter study regarding patients with MCI.

The results of our study are in keeping with previous data indicating that perfusion abnormalities play a role in the development of AD. As such, data from the brain indicate that local blood flow changes may play a role in the formation of amyloid cores in AD (Armstrong 1995). Using OCT angiography to measure perfusion in the capillary vascular bed of the retina it has been shown that the vessel density in the superficial retinal layer of patients with AD is lower compared to controls (Bulut et al. 2018). Along this line of thought, a larger study comprising 70 AD and 73 MCI eyes found that subjects with AD show a significantly reduced macular vascular density compared to MCI and cognitive healthy subjects (Yoon et al. 2019). Data from O'Bryhim and colleagues indicate that cognitively healthy individuals with preclinical AD have an increased foveal avascular zone (O'Bryhim et al. 2018). In keeping with the latter study, recently published results show that cognitively healthy individuals who are rated as preclinical stage of AD based on positron emission tomography detected Aβ aggregation show changes in vessel density and in the size of the foveal avascular zone (van de Kreeke et al. 2019b). Although OCT angiography does not provide information regarding volumetric blood flow, the studies mentioned above are compatible with the results of our study showing reduced retinal perfusion in patients with cognitive impairment.

Further, our study shows that the arteriovenous oxygen difference is reduced in the subjects of the disease group, an effect that was mainly caused by an increase in venous oxygen saturation. This has already been reported previously in patients with MCI (Olafsdottir et al. 2018) and was mainly interpreted by the authors of the study as a decrease of oxygen extraction of the retinal tissue. However, no blood flow data were available in the latter trial to support this hypothesis. Of note, oxygen saturation values observed in the current study were smaller than previously reported (Olafsdottir et al. 2018). However, the majority of studies investigating oxygen saturation in elderly were performed using a different system for the measurement of oxygen saturation, which may account at least partially for the observed differences (Told et al. 2018).

Our study extends previously published findings on oxygen saturation in patients with AD by adding volumetric blood flow data, showing a decrease in ocular blood flow in the disease group. The decrease in blood flow combined with the increase in oxygen saturation in retinal veins strongly suggests a pronounced decrease in oxygen consumption and, in turn, a strong decrease in metabolic activity (Werkmeister et al. 2015; Fondi et al. 2017; Bata et al. 2019; Stefansson et al. 2019). This hypothesis is also compatible with the structural data found in our study, showing a consistent decrease in RNFL thickness. In summary, our data are in keeping with results from studies examining the brain, where a decrease in cerebral blood flow and a decrease in metabolic activity are consistently reported in the literature (Montagne et al. 2016; Kisler et al. 2017). Interestingly, the analysis of our data does not reveal a correlation between RNFL thickness and vascular functional parameters such as blood flow or A‐V difference. Whether this is related to the limited sample size of the current study or the variability of the measurement techniques warrants further investigation. Further, it needs to be stated that both changes in RNFL thickness and oxygen are relatively small. Hence, it is currently unclear to what extend changes in RNFL thickness and/or oxygen saturation would translate or predict the clinical outcome in patients with cognitive impairment. Longitudinal studies are needed to clarify this question.

It is well accepted now that AD is associated with morphological as well as functional alterations of cerebral blood vessels and the perivascular neuronal tissue (Snyder et al. 2014). Based on these findings, it has been hypothesized that a break‐down of neurovascular coupling is an early event in ocular as well as in generalized neurodegenerative diseases in both the brain and the retina (Lourenco et al. 2015). In the current study, we have used flicker light induced hyperaemia as a widely used test to investigate whether the coupling between neural activity and blood flow is compromised (Garhofer et al. 2002; Garhofer et al. 2004; Riva et al. 2005; Kur et al. 2012; Newman 2013). Interestingly, no change in vascular response to flicker stimulation was seen in patients with AD or MCI compared to the control group. This finding is of particular interest since conflicting evidence exists regarding flicker‐induced hyperaemia in patients with AD and MCI. Kotliar and colleagues reported increased flicker response in retinal veins of patients with AD and MCI, a finding that was interpreted by the authors as either neuronal activity being increased or the feedback loop of neurovascular coupling being damaged (Kotliar et al. 2017). However, the data from Kotliar et al. are in disagreement with more recently published data: Using the same instruments for the measurement of flicker‐induced vasodilatation, Querques et al. report that patients with MCI have reduced flicker responses compared to healthy subjects (Querques et al. 2019). The reason for this difference has yet to be clarified, but may be related to differences in the patients’ characteristics between the studies.

The strength of our study is that measurement with the newly developed Doppler OCT system provides data about volumetric blood flow *in vivo*. Doppler OCT has been validated against invasive microsphere technology in primates (Told et al. 2016) and has been shown to provide blood flow information with good reproducibility. To date, the Doppler OCT system used for our measurements is not commercially available. However, although our results cannot be directly transferred into clinical routine, these data provide additional support of an altered neurovascular relationship and an impaired retinal oxygen metabolism in patients with cognitive impairment. Further, a commercial Doppler OCT prototype has been recently introduced that may soon become commercially available (Tani et al. 2017). Thus, this technique may in future be also applicable in clinical practice (Sakai et al. 2019). Another strength that needs to be emphasized is that all patients underwent an in‐depth ophthalmological examination to exclude any ocular pathologies.

However, the present study has a few limitations that need to be considered when interpreting the results. First, it is a cross‐sectional study, and therefore, it is not possible to decide how the outcome parameters change in the time course of the disease. Longitudinal studies need to be performed to finally answer this question. Second, measurements with the Doppler OCT system may take up to 20 min, require sufficient target fixation are therefore challenging for the patients, in particular for those with dementia. Thus, sufficient Doppler OCT measurements could not be obtained from all patients. Further, it needs to be mentioned that some of the patients were on antihypertensive medication. Thus, we cannot fully exclude that blood pressure medication may impact to a certain degree ocular perfusion. However, as blood pressure medication was equally distributed between the disease and the control group, this should not impact the conclusion of our study. Finally, the present study did not measure total retinal blood flow, but measurements were only obtained from one major artery and vein, respectively.

In conclusion, this study shows that retinal blood flow is reduced in patients with AD and MCI. Together with the findings of reduced arteriovenous oxygen difference, this clearly indicates reduced retinal metabolism in those patients compared to healthy subjects. No difference was observed in neurovascular coupling. Longitudinal studies are required to assess whether ocular blood flow measurements may serve as a potential biomarker to identify high‐risk patients.

## Supporting information


**Table S1.** Correlation analyses between global retinal nerve fiber layer thickness as a structural parameter with functional parameters in patients with Alzheimer’s disease/mild cognitive impairment and healthy control subjects (*Q*_A_… retinal blood flow in arteries, *Q*_V_… retinal blood flow in veins, AV‐difference… arterio‐venous difference in oxygen saturation… FL_A_… Flicker response in retinal arteries, FL_V_… Flicker response in retinal veins).
