# Supplementary material for: Anatomical and functional changes in the retina in patients with Alzheimer’s disease and mild cognitive impairment
Source: Acta Ophthalmol. 2020 Mar 25;98(7):e914–21. doi: 10.1111/aos.14419 (PMC7687124; doi:10.1111/aos.14419)
Supplement: Supplementary file 1 — Table S1. Correlation analyses between global retinal nerve fiber layer thickness as a structural parameter with functional parameters in patients with Alzheimer’s disease/mild cognitive impairment and healthy control subjects (Q A… retinal blood flow in arteries, Q V… retinal blood flow in veins, AV‐difference… arterio‐venous difference in oxygen saturation… FLA… Flicker response in retinal arteries, FLV… Flicker response in retinal veins). [file AOS-98-e914-s001.docx]

*SUPPLEMENTAL TABLE 1*

|  | **AD/MCI** | | **Healthy controls** | |
| --- | --- | --- | --- | --- |
|  | **r** | **p-value** | **r** | **p-value** |
| **Q_A_** | -0.27 | 0.15 | -0.04 | 0.84 |
| **Q_V_** | -0.05 | 0.80 | -0.07 | 0.73 |
| **AV-difference** | 0.02 | 0.89 | -0.14 | 0.42 |
| **FL_A_** | 0.24 | 0.12 | -0.05 | 0.81 |
| **FL_V_** | 0.19 | 0.21 | -0.03 | 0.86 |

*Table: Correlation analyses between global retinal nerve fiber layer thickness as a structural parameter with functional parameters in patients with Alzheimer’s disease/mild cognitive impairment and healthy control subjects (Q_A_… retinal blood flow in arteries, Q_V_… retinal blood flow in veins, AV-difference… arterio-venous difference in oxygen saturation… FL_A_… Flicker response in retinal arteries, FL_V_… Flicker response in retinal veins).*
